# Astaxanthin Extract from Shrimp (*Trachypenaeus curvirostris*) By-Products Improves Quality of Ready-to-Cook Shrimp Surimi Products during Frozen Storage at −18 °C

**DOI:** 10.3390/foods11142122

**Published:** 2022-07-17

**Authors:** Kai Zhu, Wen Yan, Zhiyuan Dai, Yiqi Zhang

**Affiliations:** Collaborative Innovation Center of Seafood Deep Processing, Key Laboratory of Aquatic Products Processing of Zhejiang Province, Institute of Seafood, Zhejiang Gongshang University, Hangzhou 310035, China; kkkaizhu@163.com (K.Z.); ywseafood@126.com (W.Y.); dzy@zjgsu.edu.cn (Z.D.)

**Keywords:** astaxanthin extract, shrimp surimi products, frozen storage, oxidative stability, color, quality

## Abstract

The effects of astaxanthin extract (AE) from shrimp by-products on the quality and sensory properties of ready-to-cook shrimp surimi products (RC-SSP) during frozen storage at −18 °C were investigated. Changes in 2-thiobarbituric acid reactive substances (TBARS) value, sulfhydryl groups, carbonyls, salt-soluble protein content, textural properties, color, and sensory quality over specific storage days were evaluated. The AE from shrimp by-products contained 4.49 μg/g tocopherol and 23.23 μg/g astaxanthin. The shrimp surimi products supplemented with 30 g/kg AE had higher redness values and greater overall acceptability and texture properties after cooking (*p* < 0.05). AE showed higher oxidative stability in RC-SSP than the control, as evidenced by lower TBARS and carbonyl content, and higher sulfhydryl and salt-soluble protein content. AE from shrimp by-products had positive effects on the antioxidant activity and color difference of RC-SSP, and could be used as a potential multifunctional additive for the development of shrimp surimi products.

## 1. Introduction

Lipids are often added during the processing of surimi-based products, due to their stabilizing effect on the network structure and flavor properties during gelation [1]. However, lipids are also easily oxidized by reactive oxygen species during storage and processing, resulting in the deterioration of product quality, which in turn affects characteristics such as taste, color, texture, and nutritional value [2,3,4]. Many studies have reported that oxidation is thought to be associated with the weakening of texture properties of surimi-based products during storage, such as gel strength and water-holding capacity [5,6,7]. Furthermore, increased levels of oxidation can also cause cellular damage, leading to severe disease in humans.

To minimize the deteriorative reaction, some synthetic antioxidants are currently being used, such as butylated hydroxyanisole (BHA) and butylated hydroxytoluene (BHT) [8]. However, they are generally toxicological and carcinogenic [9], limiting their application in the frozen food industry. Therefore, the development of natural additives for retarding protein/lipid oxidation seems justifiable. It is also a good processing strategy to extract natural antioxidant components from food processing by-products to delay the oxidation reaction in meat products. Turgut, et al. [10] reported that pomegranate peel extract was effective in preventing protein/lipid oxidation and improving the quality of frozen beef balls.

Astaxanthin is a group of xanthophylls in carotenoid, widely found in crustacean, salmon, and algae [11]. The most important biological activity of astaxanthin is its antioxidant activity. In addition, natural astaxanthin has been recognized by the United States Food and Drug Administration (USFDA) as generally recognized as safe (GRAS) [12]. Due to its strong antioxidant activity and deep red color, astaxanthin is considered a promising antioxidant and colorant [13,14]. Astaxanthin is often extracted using chemical reagents and energy-intensive methods [15,16], which may cause food safety issues and increase costs. In contrast, astaxanthin extracted from shrimp by-products by ultrasonic-assisted vegetable oil may have high food safety and processing value as a food additive [17,18]. According to Rao, et al. [19], vegetable oils can be used to stabilize astaxanthin in various applications such as food colorants, antioxidants, and nutraceutical and pharmaceutical formulations.

Ready-to-cook shrimp surimi products (RC-SSP) are made from minced shrimp or surimi and are popular among Chinese consumers for their delicious taste, rich flavor, and nutrition [20]. However, RC-SSP currently in circulation still needs improvement in terms of color and oxidative protection against lipids/proteins [21,22]. In addition, a large number of processing by-products are generated during shrimp processing, including shrimp heads, carapaces, and tails, accounting for approximately 50–60% (*w*/*w*) of the whole shrimp [23]. Although a small portion of these solid wastes have been used in the feed industry [24], most are buried or discarded, and their potential value is being ignored [25]. A recent study reported that astaxanthin had desirable effects on the antioxidant activity and color of emulsified sausages [26]. Therefore, we speculate that shrimp by-products such as astaxanthin extract may act as an effective antioxidant to maintain the oxidative stability of shrimp surimi products.

The aim of this study was to evaluate the effect of astaxanthin extract from shrimp (*Trachypenaeus curvirostris*) by-products on the oxidative stability, texture, and sensory properties of RC-SSP during frozen storage. The findings help to develop better strategies to utilize by-products of aquatic product processing and to develop functionally fortified foods.

## 2. Materials and Methods

### 2.1. Chemicals and Materials

Astaxanthin standard (purity ≥ 98%) and DPPH (2, 2-diphenyl-1-picrylhydrazyl; 98%) were purchased from Yuanye (Shanghai, China); thiobarbituric acid (TBA), 5,5′-Dithiobis-(2-nitrobenzoic acid) (DTNB) and 2,4-dinitrophenylhydrazine (DNPH) were obtained from Sigma (USA); butylated hydroxytoluene (BHT) were supplied by Aladdin (Shanghai, China).

White-hair rough shrimp (*Trachypenaeus curvirostris*) weighting 20.0–23.5 g and having a body length of 8.2–10.4 cm were purchased from Zhoushan International Fisheries City (Zhoushan, China) in June 2020 and transported in ice to laboratory within 4 h. Then, shrimp by-products (cephalothorax, shell and tail) were manually separated from the shrimp meat and lyophilized, ground, sieved through a 375-micron sieve, and stored at −30 °C for no more than 2 weeks. The shrimp meat was frozen in a −60 °C refrigerator (DW/BD-55W451EU1, Haier, Qingdao, China) for further use. Maize oil (Sanxing, Zouping, China), potato starch (83.7 g starch per 100 g dry matter basis, Saifuweng, Shanghai, China), refined salt (National Salt, Shanghai, China), and eggs (Rongda, Guangde, China) were purchased from a local market in Hangzhou, China.

### 2.2. Preparation of Astaxanthin Extract

Astaxanthin extract (AE) was prepared according to the method described by Mezzomo et al. [27] with some modifications. Briefly, three times volume of maize oil (*v*/*w*) was mixed with the shrimp by-product powder and stirred to blend well. The mixture was sonicated (410HT, Jato, Guangzhou, China) for 15 min and then incubated in a 60 °C water bath for 70 min. Finally, AE was collected by centrifugation (8000× *g*, 20 min) and stored at 4 °C for no more than 48 h.

#### 2.2.1. Determination of Astaxanthin Content

The AE was prepared by extraction with the chloroform/methanol as per the method of Mezzomo, Maestri, dos Santos, Maraschin and Ferreira [27] to determine its astaxanthin content. Briefly, the samples were mixed with 5 volumes of chloroform/methanol (1:1, *v*/*v*) solution, sonicated for 2 h, and the solvent was removed by nitrogen sweeping. Analysis was performed on a Waters HPLC system (e2695, Waters, MA, USA) equipped with a Welchrom C18 column (4.6 mm × 250 mm, 5 μm, Shanghai, China). The mobile phase was acetonitrile/methanol/water (15/80/5, *v*/*v*), the flow rate was 1.0 mL/min, and the injection volume was 20 μL. The absorbance was monitored at 487 nm. The recovery rate was calculated as the ratio of astaxanthin in AE and shrimp by-products.

#### 2.2.2. Determination of Tocopherol Content

Total tocopherol content in AE was determined according to the method described by Chaijan & Panpipat [28]. Briefly, 1 g of AE was dissolved in 1 mL of absolute ethanol, followed by the addition of 2 mL of 0.2% bathophenanthroline-ethanol and 0.2 mL of 1 mM FeCl_3_–ethanol solution in the dark. After 1 min of reaction, 0.2 mL of 1 mM H_3_PO_4_-ethanol solution was added, and the absorbance at 534 nm was measured.

#### 2.2.3. DPPH Radical Scavenging Activity

DPPH radical scavenging activity (RSA) of AE was monitored using the method described by Chintong, et al. [29]. Briefly, 1.0 mL AE was mixed with 3.0 mL of DPPH-Ethanol solution (0.06 mM), vortexed vigorously, and incubated in the dark for 30 min. The absorbance was monitored at 517 nm with ethanol as blank. The DPPH RSA was expressed as the percentage of inhibition of the DPPH according to expression: (A_0_ − A_1_)/A_0_ × 100%, where A_0_ is the absorbance of control and A_1_ is the absorbance of the sample.

### 2.3. Preparation of RC-SSP

The frozen shrimp (*Trachypenaeus curvirostris*) meat was thawed in running water for 30 min, rinsed in 25 times the volume of slurry ice (*v*/*w*), stirred for 15 min, and finally dehydrated to a moisture content of 76–82% (*w*/*w*). According to Table 1, RC-SSP was prepared using shrimp meat (895 g/kg); potato starch (50 g/kg); egg white (40 g/kg); salt (15 g/kg); and three groups of different formulations of vegetable oil: (i) 30 g/kg maize oil (control, without antioxidant), (ii) 30 g/kg astaxanthin extract (AE), and (iii) 30 g/kg maize oil containing 0.1 g/kg BHT (BHT). After all ingredients were fully mixed and chopped for 3 min, they were then vacuum packed in vacuum bags (PE/PA) and stored at −18 °C for 8 weeks. Samples were taken randomly every 2 weeks for analyses.

### 2.4. Preparation of Myofibrillar Protein

Myofibrillar protein (MP) was prepared according to Zhang et al. [8], with some modifications. Fish muscle (2 g) was homogenized (IKA T18 basic, IKA, Staufen, Germany) with 20 mL of chilled distilled water and centrifuged (Hettich ROTINA 420R, Tuttlingen, Staufen, Germany) at 8000× *g* for 10 min at 4 °C. The precipitate was dispersed in 20 mL 60 mM KCl–20 mM Tris-maleate (pH 7.0) and then centrifuged at 8000× *g* for 10 min. The obtained precipitate was further homogenized with 20 mL of 0.6 M KCl–20 mM Tris-maleate (pH 7.0), incubated at 4 °C for 1 h, and then centrifuged for 10 min. The supernatant (8 mL) was then mixed with chilled distilled water (32 mL) and centrifuged for 10 min. The precipitate was finally dissolved with 0.6 M KCl (pH 7.0). The above experiments were all carried out under 4 °C. The MP concentration was determined using the Biuret method.

### 2.5. Determination of Salt-Soluble Protein Content

The content of salt-soluble protein was determined according to Lin et al. [30]. The concentration was expressed as the protein concentration in the supernatant multiplied by the volume, divided by the mass of RC-SSP sample.

### 2.6. Determination of Thiobarbituric Acid Reactive Substances (TBARS)

TBARS values were determined according to Zhang, Dong and Dai [8] and expressed as mg malonaldehyde (MDA)/kg of shrimp surimi products. The shrimp surimi samples (5 g) were mixed with 20 mL trichloroacetic acid (TCA) solution (37.5 g TCA and 0.5 g EDTA disodium salt per 500 mL aqueous solution) in a centrifuge tube and homogenized (T18, IKA, Germany), then centrifuged for 10 min (8000× *g*, 4 °C). To start the reaction, 5 mL of the collected supernatant was mixed with 5 mL of 0.02 M thiobarbituric acid (TBA) solution, then incubated in a 90 °C water bath for 30 min. TCA solution was set as a blank. After cooling to room temperature by running water, the absorbance was monitored at 532 nm. A standard curve was calculated using 1,1,3,3-tetramethoxypropane (TEP). 

### 2.7. Determination of Sulfhydryl Groups

The sulfhydryl contents were determined using Ellman reagent according to Zhang, Dong and Dai [8]. The MP solution (0.1 mL) was mixed with 0.9 mL 8 M urea–0.2 M Tris-HCl (pH 8.0) containing 1% SDS (*w*/*w*) and 3 mM EDTA. Then, 0.1 mL of 0.1% 5,5′-dithiobis-(2-nitrobenzoic acid) (DTNB) containing 0.2 M Tris-HCl was added to initiate the reaction and incubated at 40 °C for 25 min. Absorbance was monitored at 412 nm using 0.6 M KCl–20 mM Tris-maleate buffer as a blank. The sulfhydryl group’s content was calculated using a molar extinction coefficient of 13,600 M^−1^ cm^−1^. 

### 2.8. Determination of Protein Carbonyls

The MP solutions (0.5 mL) were mixed with 0.5 mL of 10 mmol/L 2,4-dinitrophenylhydrazine (DNPH) solution containing 2 M HCl in the dark for 1 h. The reaction was terminated by adding 0.5 mL of 20% TCA (*w*/*w*). After centrifugation (8000× *g*, 4 °C) for 5 min, the precipitates were washed 3 times with ethyl acetate–ethanol solution (1:1, *v*/*v*), and then dissolved in 1.5 mL of 6 M guanidine hydrochloride containing 2 M HCl at 37 °C for 15 min. The supernatants were collected after centrifugation for 5 min. The absorbance was measured at 370 nm. The protein carbonyl content was calculated using a molar extinction coefficient of 22,000 M^−1^ cm^−1^. 

### 2.9. Preparation of Shrimp Surimi Gel

Thawed RC-SSP was cooked by two-stage heating (50 °C, 60 min; 90 °C, 30 min) and then immediately placed in ice slurry for 30 min to cool. The shrimp surimi gel was stored at 4 °C for 12 h before testing.

### 2.10. Texture Profile Analysis (TPA)

TPA was preformed using a texture analyzer (TMS-PRO, FTC, Sterling, VA, USA) equipped with a P 5S spherical probe. The test speed was 60.0 mm/s, the trigger force was 0.10 N, and the deformation was 60%. The texture characteristics of the AE group, the BHT group, and the control during storage were described in terms of gel strength, springiness, hardness, and chewiness parameters.

### 2.11. Color Assessment

A colorimeter (ColorQuest XE, HunterLab, Reston, VA, USA) was used to monitor the color difference of cooked RC-SSP during storage, where the index included L* (brightness), a* (redness), and b* (yellowness) values. In this study, redness was chosen as the primary indicator of color attractiveness for RC-SSP, because orange-red shrimp products are generally considered more attractive to consumers. 

### 2.12. Sensory Evaluation 

An evaluation panel of 20 trained members (10 males and 10 females), aged 20–40 years, selected according to American Meat Science Association guidelines, were asked to evaluate the following parameters: odor, color, umami taste, texture, and overall likeness, while a 9-pt hedonic scale was used as standard, where 9, 7, 5, 3, and 1 point corresponded to like extremely, like moderately, neither like or nor dislike, dislike moderately, and dislike extremely, respectively. To improve accuracy, the two highest and two lowest values are discarded when evaluating each item.

### 2.13. Statistical Analysis

Data were presented as mean ± standard deviation (SD). All analyses were performed in triplicate. One-way analysis of variance (ANOVA) was performed by least significant difference (LSD) with the help of SPSS 21.0 (SPSS Inc., Chicago, IL, USA) software, with a significant level *p* < 0.05. 

## 3. Results and Discussion

### 3.1. Effect of AE on Lipid Oxidation in RC-SSP

A large amount of antioxidants, such as β-cryptoxanthin, α-carotene, and phenols, have been reported to be found in the extracts derived from shrimp by-products [31]. In this study, the astaxanthin content in AE from shrimp by-products was 23.23 μg/g, and the recovery rate was 29.9%. The DPPH RSA of AE reached 79.40%, which was related to the conjugated double bonds in the molecule backbone and the terminal carbocycles with oxidized derivatives (-OH, -CO). In addition, the tocopherol content in the extracts reaching 4.49 μg/g may also contribute to the antioxidant activity of AE. Abdelmalek, et al. [32] reported that the DPPH RSA of astaxanthin-containing oils was concentration-dependent. Therefore, we speculate that the astaxanthin extract obtained by maize oil extraction may also have good antioxidant activity on shrimp surimi products.

**Figure 1:**
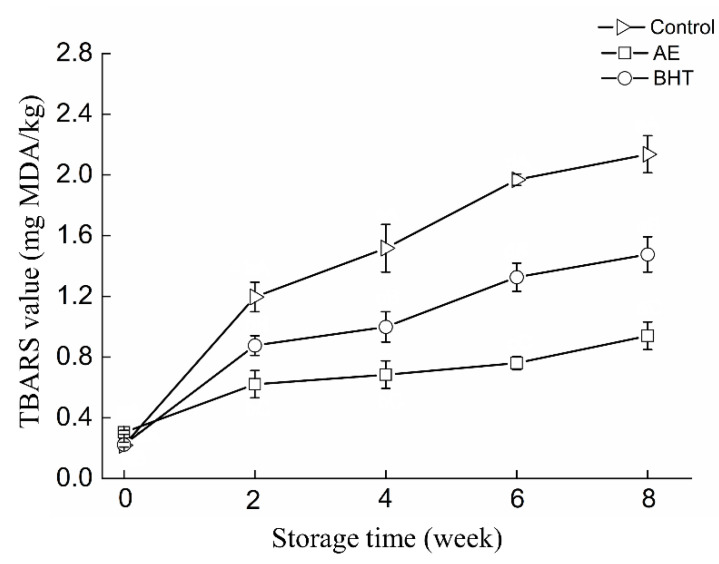
Changes in TBARS value of shrimp surimi products treated with AE, BHT, and control during frozen storage.

TBARS is a major indicator of lipid oxidation in meat and meat products [33]. As shown in Figure 1, both antioxidant treatment and storage time significantly affected the TBARS value in RC-SSP (*p* < 0.05). The initial TBARS value of the sample was 0.22 mg MDA/kg. The TBARS value of the control group increased rapidly (*p* < 0.05) with the extension of storage time, indicating that strong lipid oxidation occurred. However, in RC-SSP samples treated with AE and BHT, TBARS values increased slowly during the first 4 weeks of storage compared to control. The results showed that both AE and BHT significantly delayed lipid oxidation in RC-SSP samples during cryopreservation. Notably, the accumulated TBARS in the AE-treated samples was below 1.0 mg MDA/kg, which was considered the maximum value of fresh meat. It suggested that the addition of AE might reduce lipid deterioration by inhibiting MDA formation.

Astaxanthin has a positive effect on the oxidative stability of lipid oxidation in meat products, and the effectiveness of astaxanthin and various other natural antioxidants has been demonstrated [9,14]. Seo, Parvin, Park and Yang [26] reported that astaxanthin exhibited a similar level of inhibition of MDA formation as BHT during the storage of emulsified sausages. In the present study, AE was more effective than BHT against MDA formation in shrimp surimi products during frozen storage. Interestingly, some previous studies have shown that natural antioxidants exhibit stronger antioxidant activity than synthetic antioxidants in meat products [2,34]. Zahid, Choi, Seo, Parvin, Ko and Yang [33] reported that clove extract reduced protein and lipid oxidation (carbonyl content and TBARS value) in beef patties compared to BHT and ascorbic acid. It is currently believed that this result is due to the suppression of the production of hydroperoxides [26], but the effects of BHT and AE on free radicals need to be further investigated. 

### 3.2. Effect of AE on Protein Oxidation in RC-SSP

**Figure 2:**
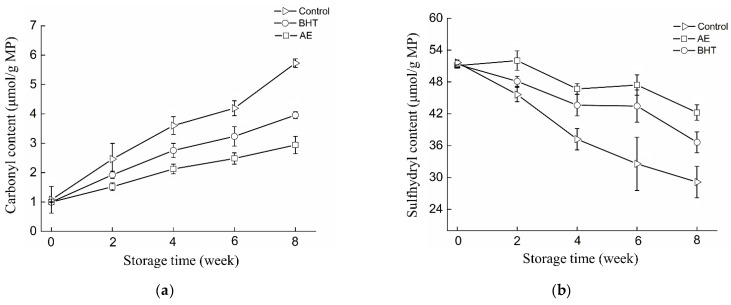
Changes in carbonyls’ contents (**a**) and sulfhydryl contents (**b**) of shrimp surimi products treated with AE, BHT, and control during frozen storage.

Protein oxidation commonly occurs in surimi-based products and can affect their texture characteristics during storage [7]. Since almost all oxidized proteins undergo carbonylation, carbonyl groups are considered reliable markers of protein oxidation. Protein carbonyls can be formed by direct modification of amino acid side chains by reactive oxygen species, peptide bond cleavage, or the addition of non-protein carbonyl units [35]. The changes in carbonyl content of shrimp surimi products during frozen storage are shown in Figure 2a. Similar to the results of the TBARS value, the carbonyl content in RC-SSP also changed with the storage time and the addition of antioxidants. In the control samples, a large amount of carbonyl derivatives accumulated, which was caused by protein oxidative modification. After 8 weeks of storage, the control had the highest carbonyl content (5.73 μmol/g MP). However, antioxidant treatment could significantly reduce carbonyl content in RC-SSP samples (*p* < 0.05). The carbonyl content of the AE-treated samples reached 2.94 μmol/g MP at the 8^th^ week, accounting for 51.4% of the control group and 74.2% of the BHT group, respectively. Similar changes in carbonyl groups in surimi protein were also reported by Lin, Hong, Zhang, Zhang and Luo [30]. 

The amount of lost sulfhydryl groups can also be used to sensitively assess the extent of MP oxidation, since myosin has a particularly high sulfhydryl group content (41 free cysteine residues per molecule) [35]. Figure 2b showed the changes in sulfhydryl content of shrimp surimi samples. It was obvious that the sulfhydryl content of RC-SSP with different treatments continued to decrease during the frozen storage period. Similar phenomenon were reported by Wang, et al. [36] and Turgut, et al. [37]. At week 8, the control samples had the lowest sulfhydryl content (29.13 μmol/g MP). However, the addition of antioxidants significantly delayed the reduction of thiol content in shrimp surimi samples. In particular, the AE group maintained the largest amount of sulfhydryl content. Loss of free thiol groups in cysteine-containing peptides and proteins readily occurs due to the sensitivity of cysteine to reactive oxygen in the presence of peroxyl radicals [38]. In this study, AE may have a protective effect on the protein structure of shrimp, preventing overexposure of buried sulfhydryl groups, thereby reducing sulfhydryl oxidation reactions in the samples [8].

The most common consequences of protein oxidation induced by free radicals include protein cross-linking by disulfides bonds, protein fragmentation, and amino acid side chain modifications (such as carbonyl formation and thiol degradation) [39]. Many natural antioxidants have a positive effect on the oxidative stability of lipids and proteins due to their chemical structure and electron-donating ability [40]. In order to delay the deterioration of food quality caused by protein oxidation, Abdelmalek, Sila, Ghlissi, Taktak, Ayadi and Bougatef [32] applied natural astaxanthin to marinated chicken steaks. Lin, Hong, Zhang, Zhang and Luo [30] reported the delayed effect of gill protein hydrolysates on the quality deterioration of frozen surimi. In this study, AE has excellent free radical scavenging activity, which can effectively delay protein oxidation. This also makes AE in shrimp by-products promising as an ingredient in the fortification of food products.

### 3.3. Effect of AE on Salt-Soluble Protein Content

**Figure 3:**
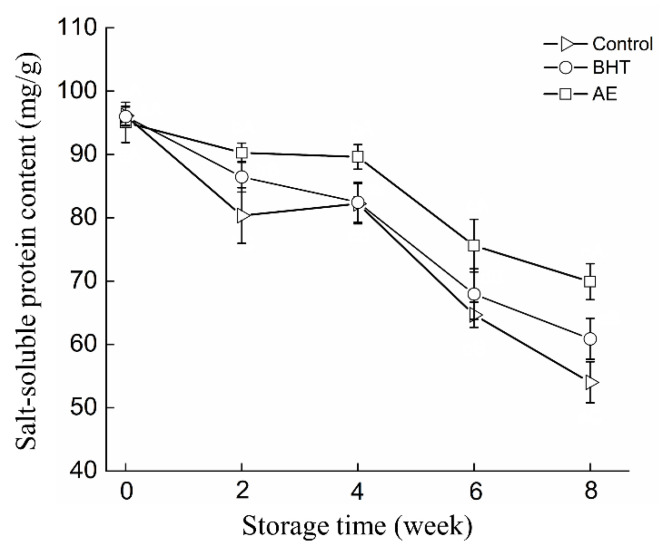
Changes of salt-soluble protein content of shrimp surimi products treated with AE, BHT, and control during frozen storage.

The salt-soluble protein content reflects the structural integrity and spatial correctness of myosin, which is critical for gel formation upon heating. The changes in salt-soluble protein content in RC-SSP treated with different treatments are shown in Figure 3. The salt-soluble protein content was highest in all groups on day 0. However, the solubility of myofibrillar protein decreased rapidly in all three groups during frozen storage, implying significant changes in protein structure. As expected, the addition of AE and BHT significantly (*p* < 0.05) delayed the decrease in salt-soluble protein content compared with the control group. The reduction of salt-soluble protein content during frozen storage was mainly due to the formation of hydrogen bonds, disulfide bonds, and hydrophobic bonds [30]. In addition to protein denaturation caused by ice crystals, protein denaturation mediated by protein oxidation has gradually attracted attention in recent years due to the general sensitivity of myosin to reactive oxygen species [7,41,42]. The results also indicated that AE could reduce oxidation-induced changes in myofibrillar protein structure. Compared with control, AE had a better protective function for myofibrillar protein within 8 weeks of frozen storage, suggesting that AE could be an effective cryoprotectant in industrial applications.

### 3.4. Effect of AE on Textural Properties

**Figure 4:**
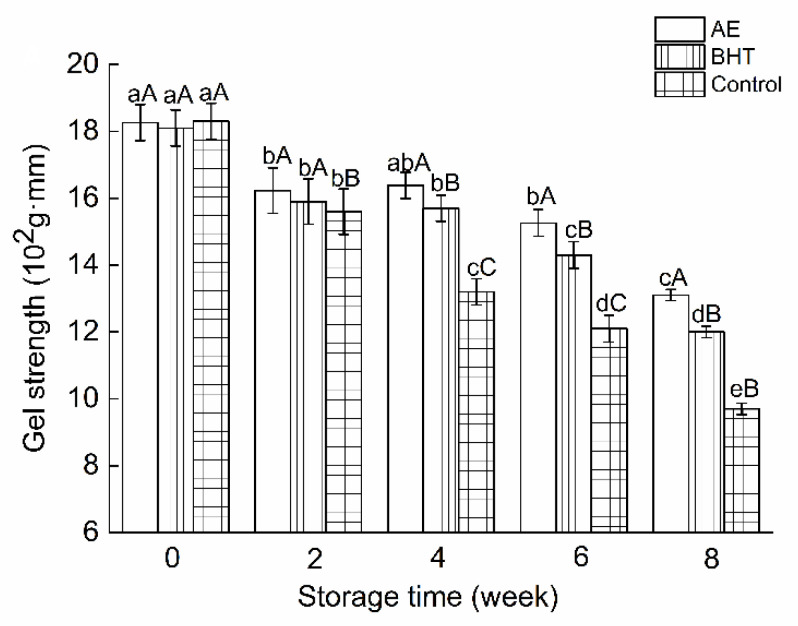
Changes in gel strength of shrimp surimi products with AE, BHT, and control during frozen storage. Different lowercase letters indicate the results that differ significantly with different storage times (*p* < 0.05). Different capital letters indicate the results that differ significantly with different treatments (*p* < 0.05).

The texture characteristics of RC-SSP affect customer acceptance. Figure 4 showed the changes in gel strength of AE and BHT-treated RC-SSP during frozen storage. The different additives did not initially show a change in gel strength. During frozen storage, the gel strength of the samples gradually decreased. Compared with the control group, RC-SSP treated with AE and BHT showed higher gel strength, and the AE-treated sample had the highest gel strength among the three groups. The results showed that the antioxidant treatment could slow down the gel deterioration during storage, and the AE showed better protection than the commercial antioxidant, BHT. 

**Figure 5:**
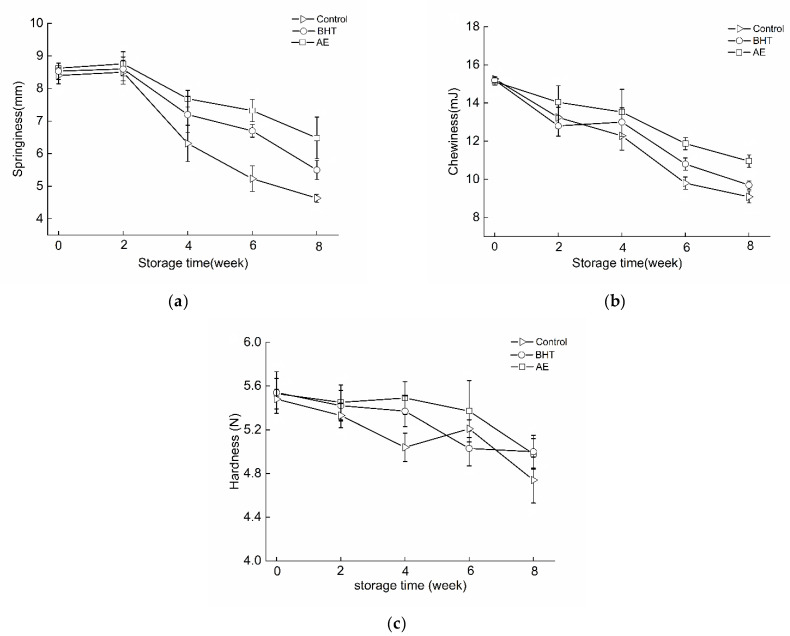
Changes in springiness (**a**), chewiness (**b**), and hardness (**c**) of shrimp surimi products with AE, BHT, and control during frozen storage.

The texture profiles of shrimp surimi products with/without antioxidants are shown in Figure 5. The springiness, chewiness, and hardness of each group showed a downward trend with the prolongation of storage time. Both antioxidant treatment and frozen storage had significant (*p* < 0.05) effects on the springiness of shrimp surimi products (Figure 5a). No significant (*p* > 0.05) difference of springiness was found among control, AE, and BHT-treated RC-SSP at initiation. Meanwhile, a slight increase was also found in all groups at week 2 and then gradually decreased, which may be highly related to the formation of disulfide bonds and the increase in hydrophobicity caused by oxidative modification on proteins [43]. After 8 weeks of frozen storage, the AE-treated RC-SSP exhibited the highest springiness, while the control group lost most of the elasticity. It can be seen that the addition of AE can alleviate the deterioration of springiness of RC-SSP during frozen storage. Similar changes were observed in chewiness and hardness, with AE-treated samples being better at maintaining textural quality (Figure 5b,c). 

The textural deterioration of RC-SSP may originate from protein oxidation and degradation [44]. Frozen storage inevitably reduced the textural properties of surimi gel due to the irregular formation and recrystallization of ice crystals [7]. In addition, myosin can be oxidized, resulting in protein-protein covalent bonding and amino acid side chain modifications [38], thereby impairing gel-forming capacity, which has received increasing attention in recent years. A previous study showed that oxidation-induced protein denaturation increased the disruption of the three-dimensional structure of pork patties after freezing, resulting in decreased texture properties [45]. Natural extracts, such as pomegranate peel [37], clove [33], and guarana seed and pitanga leaf [46], are used to prevent the textural deterioration in muscle-based gel foods through their antioxidant activity [1]. In this study, both AE- and BHT-treated samples exhibited better textural profiles than the control group. Therefore, it can be inferred that AE has a good protective effect on the texture deterioration of RC-SSP during frozen storage by inhibiting protein oxidation.

### 3.5. Effect of AE on Sensory Quality

Product color is one of the most important visual factors affecting consumer preference. Shrimp meat develops an attractive orange-red color when cooked, which is caused by thermal breakdown of the ester bonds between astaxanthin and proteins or lipids [47]. Color values of the shrimp surimi products with or without antioxidants added during frozen storage are shown in Table 2. During storage, there were significant differences (*p* < 0.05) between treatments in the color values of the surimi products such as lightness (L*), redness (a*), and yellowness (b*). The a* value of the cooked RC-SSP with AE added was higher than the control, which can be considered to be derived from the color of AE. Notably, the b* values of the AE-treated groups were also significantly (*p* < 0.05) higher than those of the control group. Previous studies have shown that yellowness is closely related to lipid oxidation, and there is often a positive correlation between b* value and TBARS and protein carbonyl content [36,48]. However, in this study, there was no direct correlation between yellowness and TBARS. The increase in b* value was more likely due to the yellow pigment in shrimp by-products. Xu, et al. [49] reported that the redness and yellowness of chitosan/gelatin composite film increased significantly after adding astaxanthin extracts derived from shrimp by-products. As shown in Figure S1, the AE exhibited a bright orange-red color, and the AE-treated RC-SSP was more attractive in color, especially redness, compared to the control. A recent study reported that the addition of 400 mg/kg astaxanthin to emulsified sausage would increase redness [26]. The AE used in this study is a mixture obtained by ultrasonic-assisted vegetable oil extraction from shrimp by-products, and its cost is much lower than that of high-purity astaxanthin. Therefore, the addition of 30 g/kg AE to shrimp surimi products would increase the redness of the product, which is also attractive in terms of cost.

**Figure 6:**
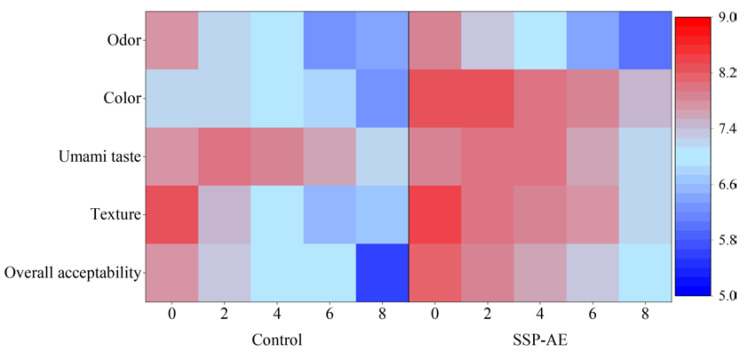
Heatmap of sensory properties of shrimp surimi products with and without astaxanthin extract during frozen storage. SSP-AE: shrimp surimi products with astaxanthin extract addition; control: shrimp surimi products with maize oil only.

To better evaluate the gel differences of shrimp surimi products caused by the addition of AE, sensory evaluations were performed in the sensory laboratory using 9-pt hedonic scale. The parameters of organoleptic properties are odor, color, umami taste, texture, and overall acceptability. As shown in Figure 6, the sensory evaluation results showed that the addition of AE had a promoting effect on RC-SSP, while all sensory scores decreased with the increase of frozen storage time. AE-treated RC-SSP had a redder color and higher texture scores and greater overall acceptability compared to the control. The AE-treated samples also exhibited higher sensory stability. The results suggested that AE derived from shrimp by-products was helpful to improve and maintain the sensory quality of RC-SSP during frozen storage. However, the effect of astaxanthin extract on the product quality during longer frozen storage needs to be further evaluated.

## 4. Conclusions

This study was conducted to investigate the effect of astaxanthin extract from shrimp by-products on the quality characteristics of ready-to-cook shrimp surimi products during frozen storage. The addition of AE could effectively retard lipid and protein oxidation in RC-SSP, as evidenced by lower TBARS and carbonyl values and higher sulfhydryl and salt-soluble protein contents. The textural properties and sensory qualities of cooked RC-SSP were effectively improved, resulting in higher texture, redness, and overall acceptability during frozen storage. The astaxanthin extract derived from shrimp by-products could be used as a potential natural multifunctional ingredient to improve the oxidative stability and sensory quality of shrimp surimi products.

## Figures and Tables

**Table 1:** Formulation of shrimp surimi products with different antioxidants.

Ingredients (g/kg)	Treatments		
	AE	BHT	Control
Shrimp meat	895	895	895
Potato starch	50	50	50
Egg white	40	40	40
Refined salt	15	15	15
Total	1000	1000	1000
Maize oil	-	30	30
AE	30	-	-
BHT	-	0.1	-

**Table 2:** Changes of L*, a*, and b* values of shrimp surimi products with and without astaxanthin extract during frozen storage ^1^.

Storage/Week	SSP-AE	Control
L*		
0	62.73 ± 0.40 ^aA^	68.03 ± 0.16 ^aB^
2	65.13 ± 0.15 ^bA^	63.62 ± 1.00 ^bB^
4	63.64 ± 0.08 ^cA^	66.66 ± 1.59 ^aB^
6	68.05 ± 0.58 ^dA^	68.00 ± 0.33 ^aA^
8	68.22 ± 0.79 ^dA^	65.00 ± 0.01 ^bB^
a*		
0	11.28 ± 0.33 ^aA^	5.33 ± 0.40 ^aB^
2	9.63 ± 0.21 ^bA^	4.79 ± 0.26 ^aB^
4	10.21 ± 0.46 ^bA^	6.80 ± 0.01 ^bB^
6	10.48 ± 0.45 ^abA^	7.69 ± 0.68 ^cB^
8	9.59 ± 0.75 ^bA^	6.23 ± 0.47 ^bB^
b*		
0	9.87 ± 0.36 ^bA^	5.76 ± 0.46 ^cB^
2	10.14 ± 0.30 ^abA^	7.34 ± 0.02 ^abB^
4	10.01 ± 0.17 ^bA^	6.43 ± 0.29 ^bcB^
6	11.20 ± 0.06 ^aA^	8.47 ± 0.97 ^aB^
8	11.19 ± 0.63 ^aA^	7.24 ± 0.98 ^bB^

^1^ SSP-AE: shrimp surimi products with astaxanthin extract addition; control: shrimp surimi products with maize oil only. Different lowercase letters indicate the results that differ significantly with different storage times (*p* < 0.05). Different capital letters indicate the results that differ significantly with different treatments (*p* < 0.05).

## Data Availability

Data are available upon request.

## Supplementary Materials

The following are available online at https://www.mdpi.com/article/10.3390/foods11142122/s1, Figure S1: Astaxanthin extract (A), shrimp surimi products with AE treatment (B) and control (C), and cooked shrimp surimi products (D).
