# Supplementary material for: Astaxanthin Extract from Shrimp (Trachypenaeus curvirostris) By-Products Improves Quality of Ready-to-Cook Shrimp Surimi Products during Frozen Storage at −18 °C
Source: Foods. 2022 Jul 17;11(14):2122. doi: 10.3390/foods11142122 (PMC9323547; doi:10.3390/foods11142122)
Supplement: Supplementary file 1 [file foods-11-02122-s001.zip › foods-1807918-supplementary.pdf]

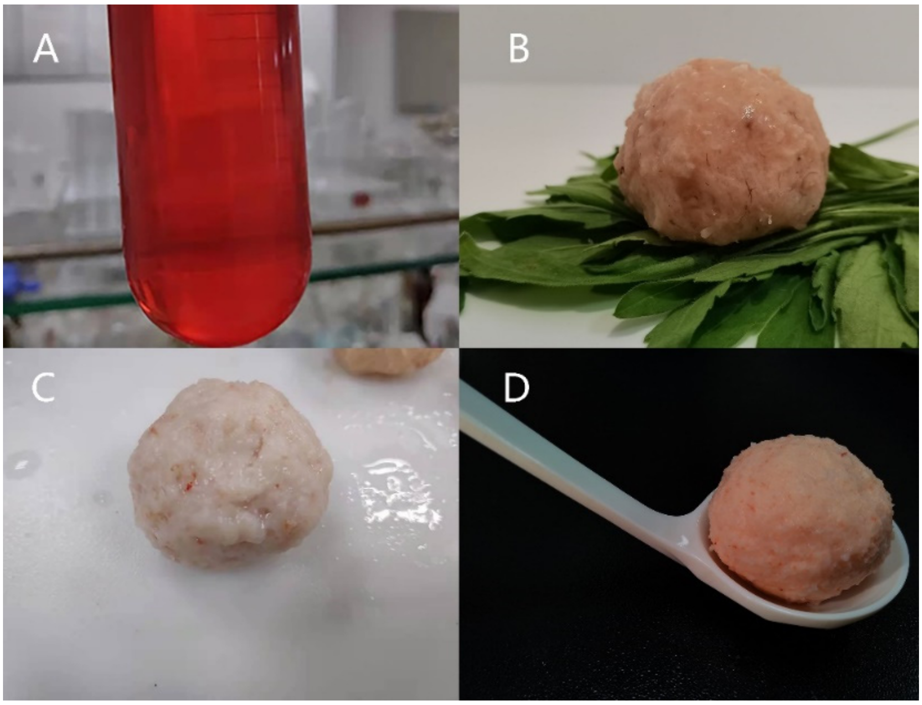

Figure S1. Astaxanthin extract (A), shrimp surimi products with AE treatment (B) and control (C), and cooked shrimp surimi products (D).
